# Toward a Trust Evaluation Mechanism in the Social Internet of Things

**DOI:** 10.3390/s17061346

**Published:** 2017-06-09

**Authors:** Nguyen Binh Truong, Hyunwoo Lee, Bob Askwith, Gyu Myoung Lee

**Affiliations:** 1 Department of Computer Science, Liverpool John Moores University, Liverpool L3 3AF, UK; n.b.truong@2015.ljmu.ac.uk (N.B.T.); r.j.askwith@ljmu.ac.uk (B.A.); 2 Media Research Division, Broadcasting & Media Research Laboratory, Electronics and Telecommunications Research Institute (ETRI), Daejeon 34129, Korea; hwlee@etri.re.kr

**Keywords:** trust, trust concept, REK trust evaluation model, Social Internet of Things, knowledge, experience, reputation

## Abstract

In the blooming era of the Internet of Things (IoT), trust has been accepted as a vital factor for provisioning secure, reliable, seamless communications and services. However, a large number of challenges still remain unsolved due to the ambiguity of the concept of trust as well as the variety of divergent trust models in different contexts. In this research, we augment the trust concept, the trust definition and provide a general conceptual model in the context of the Social IoT (SIoT) environment by breaking down all attributes influencing trust. Then, we propose a trust evaluation model called REK, comprised of the triad of trust indicators (TIs) Reputation, Experience and Knowledge. The REK model covers multi-dimensional aspects of trust by incorporating heterogeneous information from direct observation (as Knowledge TI), personal experiences (as Experience TI) to global opinions (as Reputation TI). The associated evaluation models for the three TIs are also proposed and provisioned. We then come up with an aggregation mechanism for deriving trust values as the final outcome of the REK evaluation model. We believe this article offers better understandings on trust as well as provides several prospective approaches for the trust evaluation in the SIoT environment.

## 1. Introduction

In the recent years, we have been witnessing a novel paradigm—the Internet of Things (IoT)—in which billions of electronic objects are connected to the Internet. These objects range from small and low computation capability devices such as Radio Frequency Identification tags (RFIDs) to complex ones such as smartphones, smart appliances and smart vehicles. Indeed, the idea to connect and share data among physical objects, cyber-space and humans using hyperlinks over a global network was promulgated by Lee three decades ago. A number of efforts have been made to build upon this premise in the last ten years, for example, Semantic Web (Web 3.0) integrates humans and social information to the Web, yielding a composite Cyber-Social system. With the IoT, we are now reaching to a breakthrough of a Cyber-Physical-Social System (CPSS) that connects the Cyber-Social Webs with physical world objects [1]. With billions of sensing and actuating devices deployed, the IoT is expected to observe various aspects of human life anywhere on Earth. Observation data is aggregated, processed, and analyzed into valuable knowledge describing occurrences and events regarding to different real-world phenomena. With various types of information from cyber and social domains, it is possible for a variety of services to reveal the untapped operational efficiencies and create an end-to-end feedback loop between individual’s needs and physical object responses. In order to meet the requirements for such IoT services, a unified CPSS framework has been developed that “takes a human centric and holistic view of computing by analyzing observations, knowledge, and experiences from physical, cyber, and social worlds” [2].

In the early years, most of IoT-related research articles have concentrated on RFID and Wireless Sensor Networks (WSNs) that aim at building underlying networking protocols, hardware and software components in order to enable interactions and communications among physical objects and cyber-space. However, a human-centric IoT environment in which human plays an important role in supporting application and services, are more and more perceptible. This is proven by the high rate of utilization of social phenomena and crowd intelligence when developing real-world IoT services. Consequently, the so-called Social Internet of Things (SIoT) has recently been proposed for illustrating the CPSS concept in which people are envisaged as an integral part of the IoT ecosystem [3,4]. However, the merging of physical objects, cyber components and humans in the SIoT will introduce new concerns for risks, privacy and security. Consequently, managing risk and securing the SIoT are broad in scope and pose greater challenges than the traditional privacy and security triad of integrity, confidentiality, and availability [5]. In this regard, trust is recognized as an important role in supporting both humans and services to overcome the perception of uncertainty and risk when making a decision.

Trust is a multifaceted concept used in many disciplines in human life influenced by both participators and environmental factors. It is an underlying psychological measurement to help a trustor to come up with a decision whether it should put itself into a risky situation in case a trustee turns out to be misplaced. As the aim of any SIoT services is to autonomously make decisions without human intervention, trust has been highlighted as a vital factor for establishing seamless connectivity, secure systems and reliable services. A trust platform could minimize the unexpected risks and maximize the predictability, which helps both SIoT infrastructures and services to operate in a controlled manner and to avoid unpredicted conditions and service failures.

As the importance of trust in SIoT, recently, a large number of research groups have been intensively working on trust-related areas in various networking environments such as peer-to-peer (P2P) networks, wireless sensor networks, social networks, and the IoT; varying in many applications and services from access control [6] to e-Commerce [7,8]. To develop a complete trust platform, various trust-related areas are necessarily taken into considerations such as trust evaluation and trust management [9]. In this article, we mainly focus on developing a trust evaluation model. Besides, researchers have also focused on developing trust management mechanisms dealing with trust establishment, dissemination, update and maintenance processes. Some articles have been proposed trust evaluation models based on a set of information (so-called *direct trust*) by extracting trustee’s characteristics or by observing trustee’s behaviors. This information are used to describe some trust-related characteristics of an entity that are coined as Trustworthiness Attributes (TAs); these TAs are combined to a final value for representing the trustee’s trustworthiness. The trustworthiness is then unconsciously used as trust. Other approaches have measured trust based on third-party information about a trustee that the third-parties have been already interacted with, thus, they already gained some clues of trust (so-called *indirect trust*). To do so, a mechanism needs to be created in order to evaluate opinions of an entity to another after each interaction; and to spread the opinions to others (in forms of feedback and recommendations). The final step is to aggregate the set of the third-party information to finalize an overall score which is actually the reputation of a trustee. Again, the reputation is used for quantifying trust. Reputation, which is an indicator of trust, should not be confused with trust but partially affects trust. Therefore, each of the previous research work is as a separated piece of a big picture solving a particular challenge in a specific environment.

Our on-going projects have been targeting to developing a complete platform for trust evaluation and management. The platform cooperates with applications and services to help both service consumers and providers making decisions in risky scenarios, resulting in securer activities and providing better quality of services and experiences. The platform is then considered as *Trust as a Service (TaaS)*. In this article, we aim at providing two major contributions. The first contribution is the augmentation of trust concept, definition and evaluation model that consolidate understanding on trust in the SIoT environment. This helps to remove the confusion among trust, reputation, dependability, security and privacy. The second contribution is the introduction of a complete trust evaluation mechanism in the SIoT environment called REK which comprises the three components Reputation, Experience and Knowledge. Conceptual models and evaluation approaches for the three components are proposed and described along with an aggregation mechanism for integrating the three components to finalize a trust value. An illustration for the REK model is also briefly presented using a specific use-case called User Recruitment in Mobile Crowd-Sensing (MCS) [10].

The rest of the paper is organized as follows: Section 2 provides important understandings and clarification of the trust concept in the SIoT. Section 3 describes related work as well as highlights a conceptual evaluation model with provisions. Section 4 is dedicated for describing the REK trust evaluation platform including conceptual model, prototype and the use-case. The last section concludes our work and outlines future research directions.

## 2. Augmentation of Trust Concept in the SIoT

Trust can be roughly defined as “assurance” or “confidence” of a trustor in a trustee to perform a task in a way that satisfies the trustor’s expectation. In this sense, the trustor partly recognizes the vulnerabilities and potential risks when the trustee accomplishes the task, thus it represents the trustor’s willingness to be vulnerable under the conditions of risks and interdependence [11].

### 2.1. Trust Concept Clarification

Trust is a complicated concept which was originally used in many disciplines in human life. In the SIoT environment, trust interplays between social sciences and computer science influenced by both objective and subjective factors from both participators and contextual characteristics [12].

The earliest variant of trust in computer science is system security and data security that cover concepts of hardware, software and communications. A system is trustworthy if it is secure and not compromised, meaning that it identifies people accessing the system and only allows authorized users; and the data security ensures that data is only accessed by those authorized users even in the presence of adversaries. More than three decades ago, Thomson mentioned trust in his Turing Award lecture when writing a Unix program to be free of Trojan horses [13]. Security gets further complex in networked worlds such as the Internet and the IoT due to the increasing participants to systems throughout the networks, resulting in introducing more threats, vulnerability and risks. System security and data security are also more complicated when privacy is taken into account. For example, personal data security could be ensured (in some degree) but providers can use the data for their own purposes or sell to a third-party. In this case, data security might be compromised if the data owner’s intent for data usage is violated. One of the solutions is a trust-based access control mechanism for data sharing in the environment of Smart City that we have proposed in [14].

**Figure 1:**
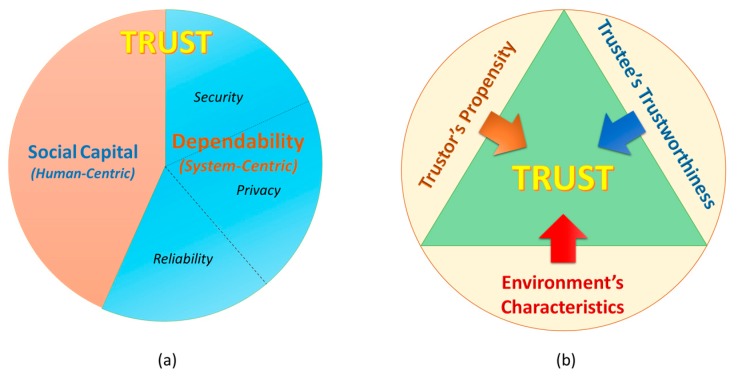
(**a**) Trust concept in the relation with dependability and social capital; (**b**) Three main aspects of trust in the SIoT environment.

An advanced variant of trust for a computer system is *dependability* that is evolved from reliability, security and privacy considerations. Besides security and privacy, reliability is a factor showing whether a systems is going to perform properly. Thus, dependability is de facto property of a system representing ability of the system to deliver secure and quality services by characterizing the security, privacy and reliability schemes in terms of some attributes such as availability, safety, integrity, confidentiality and reliability. Grandison and Sloman have defined this variant of trust as “*infrastructure trust*” [15]. In our perspective, dependability is one of the most important indicator in evaluating trustee’ trustworthiness (in case the trustee is a computer system). The key distinction between trust and dependability is due to the enrolment of social interactions (of both humans and devices), which is modulated in form of social capital factors (Figure 1a). The social capital can interpret various aspects of individuals and social networks including behaviors, norms and patterns that have built up through social interactions over time that also help to reckon trust. In this regard, trust is an umbrella concept of dependability.

Trust is originally a foundational aspect of human social relations; and when applying trust to the SIoT environment, it should be considered under a perspective of a trustor in correlation with a society. Social interactions, subjective viewpoint of individual entity, and environments should not be neglected [16]. We have pointed out that besides trustworthiness of a trustee, trustor’s propensity and environmental factors such as vulnerabilities, threats and risks also contributes to the trust evaluation (Figure 1b). This is obvious because trust only occurs risky scenarios in which the trustor is going to be under vulnerability. 

### 2.2. Definition of Trust in SIoT

There are plenty of trust definitions in particular situations resulting in difficulty in establishing a standard notation of trust in computer science. In order to define trust in the SIoT environment, we tend to follow a widely-accepted approach from social science that trust is considered as *belief* which appears in many trust-related literature [11,17]. A general definition of trust in computer science has been broadly acknowledged as following:

Trust is defined as a *belief* of a *trustor* in a *trustee* that the trustee will provide or accomplish a *trust goal* as *trustor’s expectation* within a *specific context* for a *specific period of time*.

In SIoT environment, trustors and trustees can be humans, devices, systems, applications and services. Measurement of trust as the belief (called trust value) can be absolute (e.g., probability) or relative (e.g., level of trust). The trust goal is in a broad understanding. It could be an action that the trustee is going to perform (trust for action); it could also be information that the trustee provides (trust for information). Trustor’s expectations are deliberately considered to include specific requirements for well performing (in some degree) the trust goal. All of the terms in this definition will be described and explained in detail in the next sections.

### 2.3. Trust Characteristics

Some key characteristics that further interpret the trust concept are summarized as follows:

- *Trust is subjective*: With the same trustee and trust context, trust might be different from trustors. In other word, trust is dependent on trustor’s perspective. For example, Alice (highly) trusts Bob but Charlie does not (for fulfilling a trust goal).
- *Trust is asymmetric*: Trust is a non-mutual reciprocal in nature although in some special cases, trust may be symmetric. For example, if Alice (highly) trusts Bob (in fulfilling a trust goal) it does not mean that Bob will (highly) trust Alice (in fulfilling such trust goal).
- *Trust is context-dependent*: With the same trustor and trustee, trust might be different depending on context including: (i) task goal, (ii) period of time, and (iii) environment. For instance, (i) Alice (highly) trusts Bob to provide a cloud storage service but not for a real-time streaming service; (ii) Alice (highly) trusted Bob to provide a cloud storage service two years ago but not for now; and (iii) Alice (highly) trusts Bob to provide a cloud storage service in the United Kingdom but not in the United States.
- *Trust is not necessarily transitive but propagative*: If Alice (highly) trusts Bob, and Bob (highly) trusts Charlie then it is not necessarily true that Alice will (highly) trust Charlie. However there are some evidences from the trust relationship between Bob and Charlie that Alice can rely on in order to judge the trust in Charlie.

More details about trust characteristics can be found in [18].

### 2.4. Conceptual Trust Model in SIoT Environment

**Figure 2:**
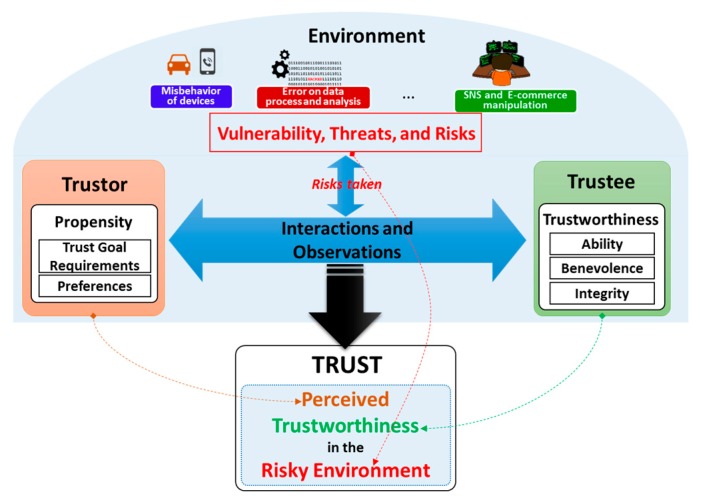
Conceptual Trust Model in the SIoT environment.

It is important to clarify that trust is neither a property of a trustor (e.g., trustor’s preferences) nor a property of a trustee (e.g., trustee’s trustworthiness and trustee’s reputation). It is a relationship between the trustor and the trustee that is subjective and asymmetric which is derived from the triad of trustee’s trustworthiness, trustor’s propensity and environment’s characteristics. Based on the clarification of the trust concept, a conceptual trust model in the SIoT is proposed as illustrated in Figure 2. Then, a more specific trust definition in the SIoT associated with the conceptual trust model is proposed as follows:

Trust is the *perception of a trustor* on *trustee’s trustworthiness* under a *particular environment* (within a *period of time*) so-called *perceived trustworthiness*.

According to the proposed model illustrated in Figure 2, trust will be obtained by harmonizing the trustor’s propensity and environment conditions into the trustee’s trustworthiness. The harmonization is accomplished by aggregating both the observation of a trustor toward a trustee and the interactions between the two. It is worth to note that the environment conditions are reflected as risks taken during the observations and interactions. The trustor’s propensity includes both requirements for the trust goal and the trustor’s preferences about the trustee’s trustworthiness whereas the environment conditions are the considerations for some factors such as vulnerabilities, threats and risks. The trust goal requirements with the environmental factors helps determining the set of TAs for deriving the perceived trustworthiness whereas the trustor’s preferences is to help combining these TAs to obtain an overall trust value for making a decision. For example, trustor’s preferences could be represented in forms of weights of TAs, indicate the levels of importance of the TAs when constructing trust. Trust as perceived trustworthiness is as an instance of trustee’s trustworthiness respecting to a particular trustor and an environment, thus, even same a trustee and same an environment, different trustors might have different propensities of the trustee’s trustworthiness. This illustrates the subjective characteristic of trust. Another important characteristic of trust is the context-dependence that can also be illustrated using this conceptual model as follows: with the same trustor and trustee, different environments might result in different TAs and different trustor’s propensities.

Based on the conceptual model, the goal of any trust model is two-fold: (i) to specify and evaluate TAs of the trustworthiness of a trustee respecting to the trustor’s propensity and the environment conditions; (ii) to combine the TAs to finalize the perceived trustworthiness as the trust value. From now on in this article, the term “trust” is referred to this conceptual model and it is exchangeably used with the term “perceived trustworthiness”.

### 2.5. Trustworthiness and Trustworthiness Attributes

According to the proposed conceptual trust model, in order to quantify trust, it is necessary to investigate trustee’s trustworthiness by specifying TAs associated with it. As mentioned above, trustworthiness is as a composite of a variety of TAs that illustrate different characteristics of the trustee. Despite a large number of TAs have been figured out in trust-related literature, TAs are mostly fallen into three categories as the three main dimensions of trustworthiness: Ability, Benevolence and Integrity. This classification is well-known and widely-accepted in the field of social organization settings [19]; and we believe it is also appropriate for consideration of trustworthiness in the SIoT environment.

- *Ability*: is a dimension of trustworthiness showing the capability of a trustee to accomplish a trust goal. An entity may be high benevolent and integrity for fulfilling a trust goal but the results may not be satisfactory if it is not capable. This term incorporates some other terms that have been used as TAs in many trust-related literature such as competence, expertness, and credibility.
- *Benevolence*: is a dimension of trustworthiness showing to what extent a trustee is willing to do good things or not harm the trustor. Benevolence ensures that the trustee will have good intentions toward the trustor. This term incorporates some TAs such as credibility, relevance, and assurance as TAs.
- *Integrity*: is a dimension of trustworthiness showing the trustee adheres to a set of principles that helps the trustor believe that the trustee is not harmful and not betray what it has committed to do. These principles can come from various sources such as fairness, or morality. This term incorporates some TAs such as honesty, completeness, and consistency.

Table 1 lists a miscellany of TAs keywords classified into the three categories. Some of the TAs in Table 1 are frequently used in trust literature ranging from social science to computer science, the other are rarely used and only existed in specific contexts. Even though each of the three factors Ability, Benevolence and Integrity captures some unique elements of trustworthiness, many of these keywords are not necessarily separated, and the interpretations of them clearly depend on particular environments and trust goals. For some specific environments and goals, certain TAs are similar whereas they are different in other contexts.

## 3. Trust Evaluation Model: Background and Provisions

Trust can only be measured partly. It is impossible to measure trust completely due to a huge range of factors from both participants and environment contributing to the trust relationship. Moreover, some of them are unable to obtain or greatly challenged to measure.

### 3.1. Brief Understanding on How to Evaluate Trust

As implied in the conceptual model in Section 2.4, a trivial trust evaluation scheme could be as the following procedure: (i) determine and calculate all TAs of a trustee’s trustworthiness; (ii) specify task requirements and preferences, (iii) figure out all environment conditions; then (iv) incorporate these factors to build trust. This trust evaluation model is called *direct trust* that indeed calculates trust based on direct observations on both the participants (the trustor and the trustee) and the environment. However, this approach finds unfeasible to efficiently measure trust due to several reasons. For example, there are variety of TAs (some of them are listed in Table 1) need to be quantified in order to measure the *direct trust*; and this is an impossible mission. One reason for this is due to the ambiguity and variability of natural language when defining terms for TAs that are still debatable in trust literature. Another reason is the complication and limitation of data collection, technologies and methodologies for valuating all the TAs as well as the complexity of incorporating TAs with trustor’s propensity and environment conditions to evaluate trust. Authors in [20] also mentioned that TA collection might cause privacy leakage which makes involved entities reluctant to provide personal evidence for a trust evaluation platform.

**Figure 3:**
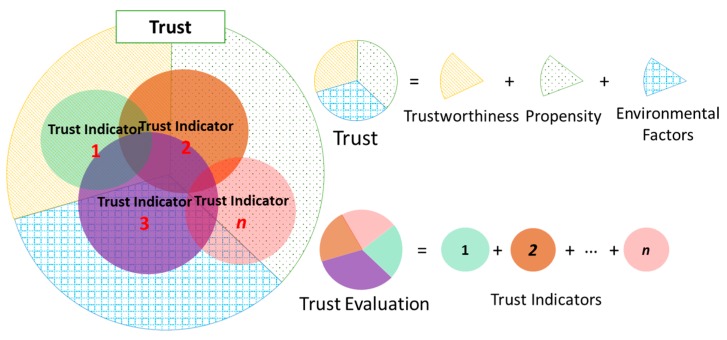
Concept of computational trust that comprised of multiple trust metrics.

Consequently, instead of measuring trust using only the direct trust approach, a prospective approach is to determine a set of indicators called Trust Indicators (TIs) that are feasible, not so complicated to obtain, and cover different aspects of trust. As the word “indicator” implies, each TI is as a “piece of a puzzle” showing the consensus of trust. TIs could be a TA or a combination of several TAs; could also be a combination some TAs with trustor’s propensity and environmental factors. TIs can be obtained using different approaches, for instance, the direct trust evaluation model could produce a good TI. However, other TIs do not necessarily only stick to the direct trust evaluation scheme. Thanks to the integration of social networks, some TIs can be determined based on social interactions in the SIoT environment that effectively indicate trust such as Recommendation and Reputation which are evaluated contingent on the propagation characteristic of trust. These TIs are then combined to derive a portion of the *complete trust* called *computational trust*. The *computational trust* is persuasively used on behalf of the *complete trust* (Figure 3). As many TIs are specified and evaluated as more accurate the *computational trust* will get. However, as two sides of a coin, there is always trade-off between computational trust accuracy and computational efforts.

Nevertheless, any trust evaluation models in SIoT environment should determine two objectives: (i) specify a set of TIs in which each TI represents a piece of the three factors: trustee’s trustworthiness, the trustor’s propensity, and the environmental factor; (ii) propose mechanisms to evaluate the TIs as well as to derive the computational trust value from the TIs. Again, the *computational trust* should be much similar to the *complete trust* so that it can be efficiently used on behalf of the *complete trust* in most of the cases.

### 3.2. Related Work on Trust Evaluation

Despite the importance of trust in computer science, there are limited notable articles that clearly clarify the trust concept, trust models and evaluation mechanisms, especially in the IoT environment. A variety of models and mechanisms have been proposed for evaluating trust, however, they have mainly focused on building reputation systems in social networks for e-Commerce services [21,22] or focused on developing trust management mechanisms in distributed systems such as WSNs [23,24], mobile ad-hoc networks (MANET) [25,26,27], and P2P networks [6,28]. The trust evaluation mechanisms in these articles are mostly based on insufficient information (i.e., only direct observation information or only third-party information).

Some trust models attempt to assess trustee’s trustworthiness by introducing some TAs and associated evaluation mechanisms for generating a so-called trust. They indeed calculate *direct trust* that is a portion of the perceived trustworthiness. Researchers have pointed out that in some scenarios such as MANETs, due to high mobility, it is challenged to maintain a centralized system for managing third-party information, resulting in only direct observation information is possibly obtained; and they have to adapt the trust models based on constrains of the environments [25,26]. In these evaluation models, the *direct trust* consists of a set of manifold TAs that are necessary and sufficient for a trustor to quantify trust in a particular environment. The perceived trustworthiness is not required to cover all TAs, instead, the set of TAs should be deliberately chosen based on the trustor’s propensity and the environmental factors (even though in these articles, the trustor’s propensity and the environment characteristics are not mentioned). For example, when evaluating trustworthiness of sensor nodes in WSNs, Bao and Chen have used Cooperativeness, Community-Interest, and Honesty to judge whether a sensor node is malicious or not. These TAs help to evaluate trustworthiness of a sensor node in a WSN that contains some types of vulnerabilities and attacks [23]. The disadvantage of this approach is that the authors do not have a mechanism to combine such information to illustrate the subjectivity of trust. Thus, what they calculate is as an instance of entity’s trustworthiness. Y. Yu et al. in [24] have analyzed various types of threats and attacks and variety of trust models in the WSN environment for secure routing protocols by characterizing many attributes of a secure system such as security mechanisms and attack preventing mechanisms. Li et al. in [27] have used only local information about a node for evaluating trust, giving an incomplete partial trust for a trust management called Objective Trust Management Framework (OTMF) in MANETs environment. The novel idea is that they apply a modified Bayesian model using different weights assigned for each information obtained from direct observations. The information is collected using a watchdog mechanism; and in order to calculate weights for each kind of information, the OTMF floods all the observation information throughout the network. A node can rely on the observation from neighbors (called second-hand information) for determining its own weights. The problem of the mechanism is the generation of a significant amount of overhead to MANETs. In [6,29], the authors have mentioned about trust-related information extracted from the three layers of a networking system namely physical, core and application layers; and they use the information for quantifying trust. An inference engine based on fuzzy logics is used to infer a trust level. However, the drawback of this approach is only focusing on objective factors only but not subjective factors of trust. As a result, values they got from the computation mechanism do not reflect some key characteristics of trust, thus cannot quantify as trust. An interesting article is about judging trust based on several features extracted from social interactions such as spatiality, relative orientation, frequency of interactions, and duration of interactions [30]. However, this information is not sufficient to accurately derive trust due to a variety of assumptions on relations between trust and behaviors of entities which are sometimes not correct.

Some trust models imitate the human cognitive process to form a belief value by considering several types of TIs such as reputation and recommendation and observation. These models have been proposed for trust evaluation and trust management in P2P networks [31], Social Networks [32], IoT [23,33] and in SIoT [34]. Most of them are based on interactions among entities in (social) networks to evaluate trust, resulting in a distributed, activity-based or encounter-based computation model. Here, trust is derived only based on social concepts such as reputation, recommendation and experience by propagating knowledge among entities. Reputation has been widely used in many applications and e-Commerce websites such as eBay, Amazon, and IMDb, however, the biggest drawback of these reputation schemes are the requirements of human participants in giving feedback as their opinions about the entities they have interacted with. In addition to the online transactions in e-Commerce, reputation schemes can be used in purely P2P, MANETs and WSNs systems that facilitate interactions among entities distributed over a network. For instance, many trust-based routing protocols in WSNs and MANETs assess trustworthiness of a node in the networks by considering third-party opinions and reputation as well as their own experiences based on their understanding to make sure that a node is not going to be misbehavioured and compromised. Based on the trustworthiness value, a decision maker will choose whether the node is put into routing paths or not. For example, a time-sensitive and context-dependent trust scheme in MANET is proposed as a combination of self-measurement and neighbor sensing (as recommendation) for enhancing trust evaluation accuracy [35]. Nitti et al. in [34] have also proposed a trust management scheme in the SIoT that incorporates several TIs extracted from feedbacks such as credibility, relationship factors, and transaction factors; as well as incorporates some TIs from direct knowledge such as computational capabilities showing the potentiality of an object to damage other objects.

Another notion of trust is ranks among webpages introduced by Google in their PageRank^TM^ mechanism [8]. In this example, webpages are listed in descending orders of levels of trust of the trust between a user and a webpage. The trust goal in this case is that the webpages should be the correct targets the user is searching for. The mechanism actually assesses a composite of reputation and importance of a webpage by observing network behaviors with an assumption that “the more back-links to a webpage, the more reputation and importance it gets (and higher probability users will visit such webpage)”. In this sense, PageRank^TM^ value is partial trustworthiness of a webpage and it is used as a TI. Even though PageRank^TM^ is just a portion of trust and does not carry some important characteristics (e.g., subjectiveness and transitivity); in this webpage ranking scenario, it is effectively used on behalf of trust.

### 3.3. Trust Evaluation Versus Risk Management

Apart from the main content of the article, it is worth to mention the correlation between trust evaluation and risk management due to the need for assessing risk (in some degree) as environmental factors when evaluating trust. Managing risk for a computer system is a complex and multifaceted process including: (i) frame risk; (ii) assess risk; (iii) respond to risk once determined; and (iv) monitor risk. These four tasks require a full investigation of vulnerabilities, threats and risks in networking systems [36].

**Figure 4:**
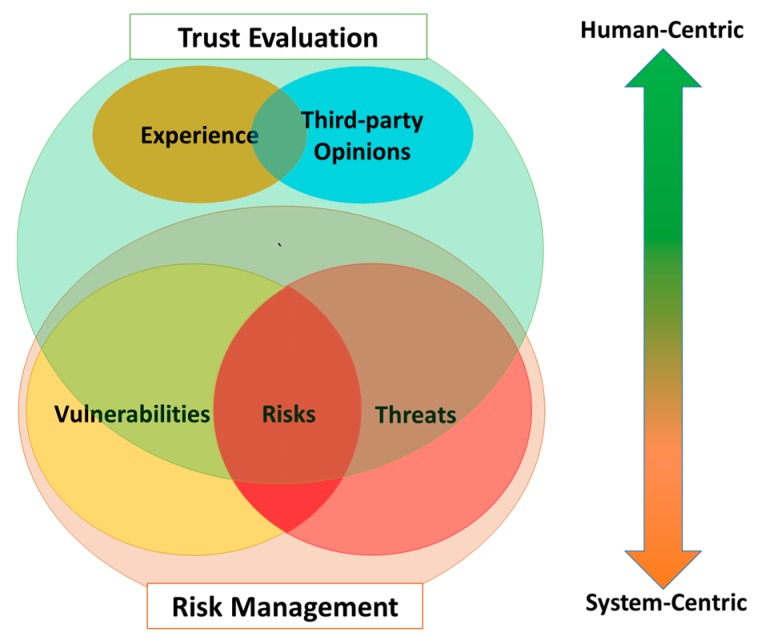
Trust evaluation and risk management in comparison.

The analysis of vulnerabilities, threats, and risks is also required in the trust evaluation but it is not necessarily fully involved as in the risk management. Instead, trust evaluation takes social-related factors (i.e., Experience and Third-party Opinions) into account when judging trust (Figure 4). Risk management assesses an entity (i.e., a computer system) from the perspective of a system (system-centric) while trust considers the entity (the trustee) under perspectives of a trustor, expressing a subjective view of the trustor on the trustee in an associated social context (human-centric).

## 4. REK Trust Evaluation Model in the SIoT

### 4.1. REK Trust Evaluation Model

**Figure 5:**
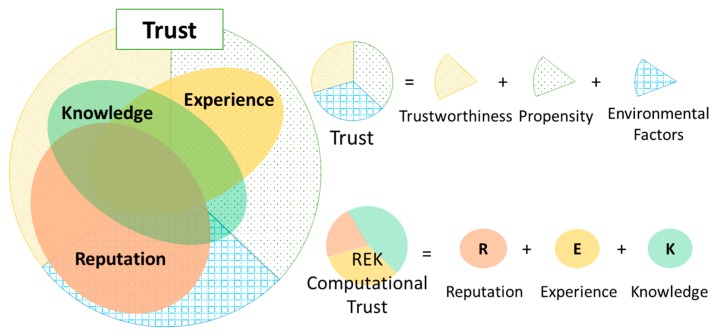
Reputation, experience and knowledge as the three indicators in the REK trust evaluation model.

We propose a trust evaluation model that comprises of triad of Reputation, Experience and Knowledge TIs so-called REK Trust Evaluation Model (Figure 5). The reason to come up with the three TIs is that in social science, people normally base their determination of trust on three main sources: (i) public opinion on a trustee (as Reputation); (ii) previous transaction with a trustee (as Experience); and (iii) understandings on a trustee (as Knowledge). We believe this social cognitive process could be applied to the SIoT environment.

Knowledge TI is the *direct trust* mentioned in Section 3 that renders trustor’s perspective on trustee’s trustworthiness in a respective environment. Knowledge TI can be obtained based on limited available information about characteristics of the trustee and the environment under the trustor’s observation. Knowledge TI can reveal a portion of trust which is illustrated in Figure 5. It indicates more about trustworthiness of the trustee and trustor’s propensity but not much about the environmental vulnerabilities, threats and risks.

Experience and Reputation TIs are social features and attained by accumulating previous interactions among entities in the SIoT over time. Experience TI is a personal perception of the trustee’s trustworthiness by analyzing previous interactions from a specific trustor to a particular trustee in various contexts. As the personal perception, Experience TI indicates more about trustor’s propensity but not trustee’s trustworthiness and environmental factors due to limited knowledge obtained. Reputation TI, instead, reflects global perception about a trustee by aggregating all previous experiences from entities (in a society) with this trustee. Thus, Reputation TI is able to effectively exhibit about the trustee’s trustworthiness and the environment characteristics; but not about the trustor’s propensity (Figure 5). In SIoT scenarios with billions of entities, there is very high possibility that there are no prior interactions between two any entities, resulting in no Experience. Therefore, Reputation TI is a necessary indicator for trust, especially in case there are no previous interactions between a trustor and a trustee. Reputation is taken into account when evaluating trust because of the propagation characteristic of trust: Each entity (a trustor) has previous interactions with a specific entity (as the trustee) has its own opinions; and a reputation model (or a recommendation model) let it share the opinions (as its recommendations) to others. Entities, then, can refer the opinions as one of the cues of trust to personally judge trust. By doing so, trust is propagated throughout the network.

By synthesizing the three TIs, REK Trust Evaluation Model consolidates the *computational trust* so that it can be used on behalf of the *complete trust* in most of cases in the SIoT environment with high accuracy.

### 4.2. Knowledge TI Evaluation Model

Knowledge TI unfolds perception of a trustor toward a trustee about how trustworthy it accomplishes a trust goal in a specific context in SIoT. It leverages the direct trust evaluation model mentioned in Section 3, thus, comprises of two major tasks: (i) specify a set of TAs for the trustee’s trustworthiness that reflects the trustor’s propensity and the environmental factors; and (ii) an aggregation mechanism to combine these TAs for deriving the *direct trust* as the Knowledge TI value. In this section, a general TAs set is introduced which covers sufficient information to evaluate *direct trust* in the SIoT environment; then, a TAs set for the specific use-case User Recruitment in MCS is specified and described as the detailed illustration for the general TAs set. The second task will be clarified in Section 4.4.

#### 4.2.1. A General Set of TAs for Knowledge TI

**Figure 6:**
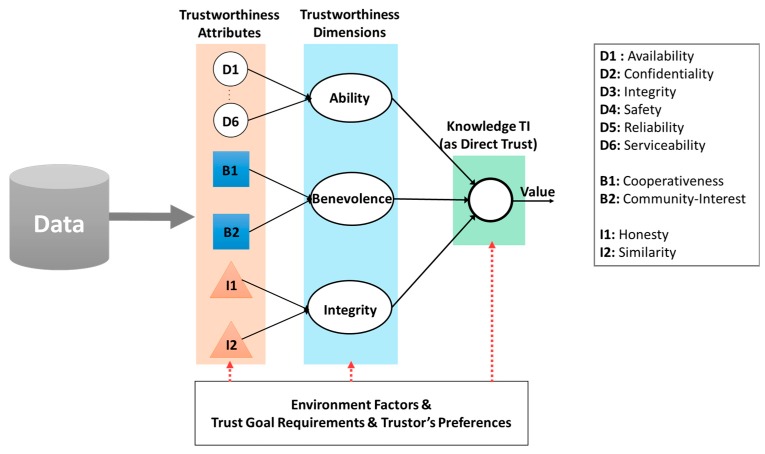
Evaluation model for direct trust (as Knowledge TI).

For the first task, we specify six important attributes introduced in the system dependability concept namely Serviceability, Safety, Reliability, Confidentiality, Availability, and Integrity as six TAs for the *Ability dimension* of the trustworthiness illustrated as D1 to D6 in Figure 6. These six TAs could precisely indicate capability of a trustee to dependably accomplish a trust goal. Besides, the *Ability dimension* might contain other TAs according to a specific scenario. For instance, in the User Recruitment in MCS use-case, the spatial distance between a trustor and a trustee is considered as a TA (see Section 4.2.2). The meanings of the six TAs in quantifying trustworthiness are as following:

- *Availability*: Probability of an entity in operation in a given period of time.
- *Confidentiality*: Preserving the authorized restriction on access and disclosure on data, information or system.
- *Integrity*: Ability to guard against improper modifications and destruction.
- *Safety*: A property to guarantee that an entity will not fail in a manner that would cause a great amount damage in a period of time.
- *Reliability*: Probability that a component correctly performs a required job in a specified period of time under stated conditions.
- *Serviceability*: Property indicating how easy and simply a system can be repaired or maintained.

Generally, combination of the TAs is a measure of a system’s capability to accomplish a given task that can be defensibly trusted within a period of time [37]. However, it is not necessary to include all of the six TAs which could require huge effort. Instead, only some of them are necessarily taken into consideration according to a specific trust goal and environmental factors. The TAs are quantitatively or qualitatively measured based on different types of information and methodologies, which have been intensively explored over time [38]. Each TA can be slightly interpreted and attained differently depending on particular use-cases due to the variations and ambiguity of its linguistic meaning. Details of dependability models can be found on a large number of articles such as Cyber-Physical System (CPS) Framework [39] and Managing Information Security Risk [36] by National Institute of Standards and Technologies (NIST).

As SIoT environment, we characterize two major TAs constituted the *Benevolence dimension* for Knowledge TI as *Cooperativeness* and *Community-Interest* illustrated as B1 and B2; and two TAs constituted the *Integrity dimension* as *Honesty* and *Similarity*, illustrated as I1 and I2 in Figure 6, respectively.

- *Cooperativeness*: this property indicates the level of cooperativeness between a trustor and a trustee based on the following hypothesis: “the more cooperative between the two entities in a social network, the more trustworthy they are”. Cooperativeness can be calculated by considering the common features between the two entities such as mutual friends and same locations.
- *Community-Interest*: Due to the integration of social networks in SIoT, concept of community (of SIoT entities) is also introduced that refers to a group of entities sharing same characteristics (e.g., physical areas, a same goal, and same required tasks). This property indicates the level of community relationship between two entities based on the following hypothesis: “the more similar among communities that entities belong to, the more trustworthy they get”.
- *Honesty*: a property indicates the level of honesty of an entity based on observation toward an entity that whether it conducts some suspicious interactions or it breaks social etiquette using a set of anomaly detection rules.
- *Similarity*: a property indicates the level of similarity between two entities (in terms of their features) using similarity measurement mechanisms between two profiles of entities [40]. This TA is taken into account because of the following hypothesis: “a trustor tends to trust a trustee if they are similar”.

These four factors are chosen to determine an entity in a society which is trustworthy or malicious; and also to recognize the SIoT environment risks including various types of attacks in social networks such as self-promoting, bad mouthing, and ballot stuffing [41]. Therefore, the combination of these four TAs guarantee to explicitly indicate whether an entity is trustworthy in a social network or not. By integrating the Ability, a perceived trustworthiness in the SIoT environment could be effectively achieved.

#### 4.2.2. User Recruitment in Mobile Crowd-Sensing Use-Case

**Figure 7:**
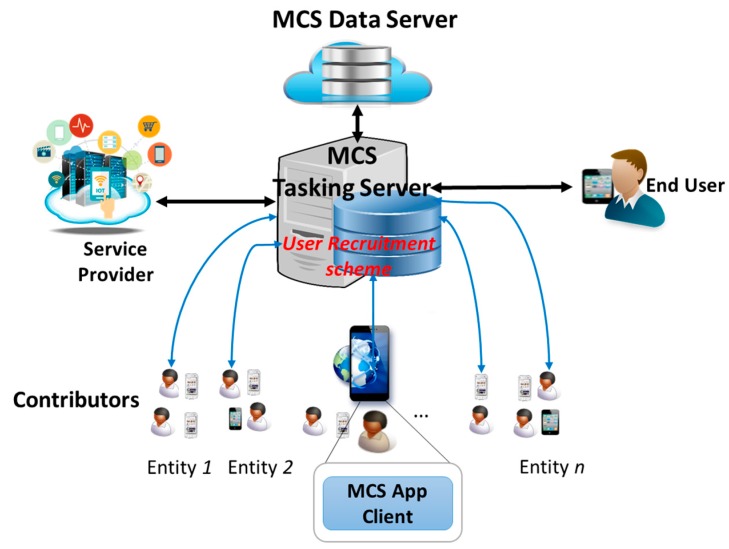
Mobile Crowd-Sensing System Architecture.

Most of applications and services in IoT heavily depend on massive amount of data collected from various types of sensors. However, traditional sensor network schemes have never reached to full potential or successfully deployed in the real world due to high installation cost, insufficient spatial coverage and so on. As a prospective solution for the traditional sensor networks, recently, the new sensing paradigm MCS has attracted attentions from both academia and industry [10]. MCS is a large scale sensing mechanism leveraging smart devices integrated with built-in sensors such as mobile phones, tablets, wearable devices and smart vehicles. It expands the traditional participatory sensing by involving both participatory sensory data from devices and social information from mobile social networking services [42]. MCS offers a large number of mobile sensing devices owners to share knowledge (e.g., local information, ambient context, noise level, and traffic conditions) acquired from their devices which further aggregated in cloud for large-scale sensing and intelligent mining [43] (Figure 7), thus enables a broad range of applications such as traffic planning, public safety, environment monitoring, and mobile social recommendation.

One of the challenges in MCS is the recruitment of contributors for sensing tasks [44,45]. In a crowded urban area with high number of participants, it is critical to recruit trustworthy users to collect high quality of data as well as to guarantee security, privacy and data integrity. This challenge calls for an efficient User Recruitment scheme implemented in the MCS Tasking Server for making proper selection of contributors respecting to a specific sensing task as illustrated in Figure 7 (the sensing task requested by service providers and assigned based on a mechanism deployed at the MCS [46]). Note that in order to recruit users evolving in a sensing task, the MCS Tasking Server should manage an incentive scheme as rewards for their contributions because users sustain costs (e.g., energy consumption, data subscription, and privacy and security breach) for accomplishing assigned sensing tasks. The User Recruitment scheme specifies criteria for user eligibility to contribute to a crowd-sensing campaign by judging whether a user accomplishes a sensing task as expected. In other words, the MCS Tasking Server chooses contributors as it trusts to fulfil the sensing task. Therefore, this use-case turns to a trust scenario as follows:

Evaluate trust between the MCS Tasking Server (as the trustor) and owners of mobile devices (as the trustees), respecting to a sensing task (as the trust goal).

A sensing task called Traffic Congestion and Accident Report is considered as follows: Report accidents and traffic congestion at a specific crossroad X. The sensing task is event-based, spatial, urgent, and nearly real-time required. Contributors should report situation of the traffic situation at the crossroad X by sending data obtained from smartphone sensors such as accelerometer, magnetometer, and GPS coordinates as well as submitting an image or a video about the traffic incidents [47,48]. Based on the proposed Knowledge TI model, a set of TAs is deliberately chosen as following:

- *Spatial Distance*: This TA shows the distance between a contributor and the crossroad X. The contributors should be close enough to the crossroad X so that it is able to report traffic situation correctly to the MCS server. The distance can be calculated based on the GPS coordinates of the smartphone and the crossroad X using the “*haversine*” formula presented in [49]. This TA belongs to the *Ability dimension* and should not exceed the distance boundary (as a threshold).
- *Availability*: Availability is a TA indicating the activeness of a user in getting connected to social activities. It shows how much a user uses his smart device for social applications and is ready to fulfil an assign task which is essential to consider for user recruitment. The Availability can be calculated based on both time spending on social network application and amount of data consumed [44,45]. This TA belongs to the *Ability dimension*.
- *Transmission Capability*: It is required to be reliable, fast, and secure when fulfilling important tasks in traffic incident reports; thus this indicator is essential for reflecting the capability of a smart device to transmit data in real-time or nearly real-time as well as in a secure and privacy manner without compromise. Therefore, this indicator includes several TAs in Ability dimension mentioned in Section 4.2.1 such as Reliability, Confidentiality and Integrity. For simplicity, we specify the level of the Transmission Capability based on some information: *signal strength*, *signal-to-interference-plus-noise-ratio* (*SIRN*), and the *communication technology* in use (*WiFi, LTE, 3G, WiMax*, and *Bluetooth*). For example, Transmission Capability is *high* when the user is using *4G LTE* for data transmission with high signal strength (*4G LTE Signal ≥ −50 dBm*) and *high LTE SIRN (LTE SIRN ≥ 12.5)* whereas it is *low* when *3G* is used with *low 3G SIRN (3G SIRN ≤ −5).*
- *Cooperativeness*: This TA represents the degree of a user cooperates with crowd-sensing tasks, thus, high cooperativeness indicates more opportunities that the user is willing to accomplish an assigned sensing task, and vice versa. This TA belongs to the *Benevolence dimension*. Cooperativeness can be simply calculated by using Equation (1):
  (1) $$Cooperativeness\left(i\right)=Frequency\left(i\right)\times \frac{\left|Number\;of\;tasks\;involved\right|}{\left|Number\;of\;tasks\;requested\right|}$$
  where Frequency(i) indicates how frequently the user *i* has involved in the crowd-sensing campaign. It is calculated based on Equation (2)
  (2) $$Frequency\left(i\right)=\frac{\left|Number\;of\;sensing\;tasks\;involved\right|}{\left|sampling\;period\;of\;time\right|}$$
  The numbers of tasks requested is the number of times the MCS Tasking server has requested the user to participate in a sensing task; and the number of tasks involved is the number of times the user has accepted to involve in sensing tasks that the MCS has requested. The number of tasks canceled is the number of times the user cancels a sensing task when it has already accepted to involve in the sensing task. The number of requested, involved, and canceled sensing tasks of the user *i* is kept track and managed by the MCS Tasking Server.
- *Honesty*: This TA represents the degree of keeping promise once a sensing task is already assigned to a user. High honesty means that the user is not going to cancel a task once it is assigned due to any cause whatsoever. This TA belongs to the *Integrity dimension* and it is simply measured by the Equation (3).
  (3) $$Honesty\left(i\right)=1-\frac{\left|Number\;of\;tasks\;canceled\right|}{\left|Number\;of\;tasks\;involved\right|}$$

An aggregation mechanism for inferring the direct trust Knowledge TI will be prototyped in Section 4.5.

### 4.3. Experience TI Evaluation Model

**Figure 8:**
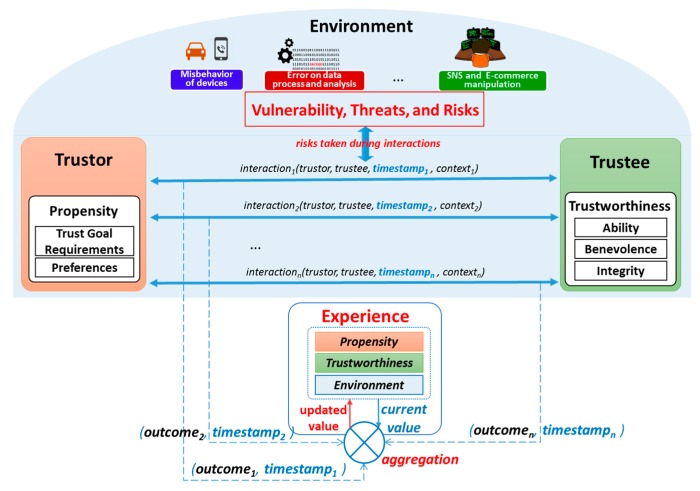
The experience TI model in the REK trust evaluation.

Experience is a social concept that represents personal understandings and opinions about one entity to another based on its previous interactions to the counterpart. We propose a conceptual model for the Experience TI depicted in Figure 8 which computes experiences based on the three factors: the current value of Experience, the outcomes, and the timestamps of individual interactions. Therefore, an outcome evaluation scheme for the interactions is one of the important components in the Experience TI model. Various mechanisms can be used to deduce outcomes of interactions depending on particular scenarios. For instance, outcomes might be feedback (in both implicit and explicit forms) from consumers after each interaction (as used in many e-Commerce and reputation systems), might just be a Boolean value (or 0/1) generated by using an ACK message to track whether the interaction has successfully accomplished or not (as in some reputation-based trust systems). For example, in Wireless Sensor Networks, interactions are package transmissions between two nodes, if a transmission is successful, then the outcome of the interaction is 1, and 0 otherwise. In a file-sharing P2P networks, interactions are file transfer transactions. If a file is successfully transferred, then the outcome of the interaction is 1; otherwise is 0. The interaction is also in form of any types of relationship between two entities. For example, Google PageRank^TM^ considers a hyperlink as an interaction between a source webpage and a destination webpage; and the outcome value is set as 1 [8].

Another important component is an aggregation model for calculating Experience TI. There is an important assumption about experience relationship between humans in sociological environment: Experience accumulates for cooperative interactions and is decreased by uncooperative interactions. It also tends to decay over time if it is not maintained by interactions. This assumption has been reasonably proven in many trust-related sociological literatures [50,51]. Thus, there are three trends of the experience relationship: Increase, Decrease, and Decay; and all of them are measured based on three features: *intensity of interactions*, *values of the interactions*, as well as *the current value of the experience*. Therefore, a mathematical linear difference equation could be used to model the trends of the Experience TI. We have proposed an Experience TI model in which an outcome of an interaction is either 0 (indicates uncooperative interaction) or 1 (indicates the cooperative interaction). The model consists of three trends is proposed as following:

- *Experience Increase (in case of a cooperative interaction occurs)*:
  The Experience Increase trend is modelled using a linear difference equation as following:
  (4) $$Experience_{t+1}=Experience_{t}+\Delta Experience_{t+1}$$
  (5) $$\mathrm{where}\;\Delta Experience_{t+1}=\alpha -\frac{\alpha }{max_{Experience}}\times Experience_{t}$$
  where $Experience_{t}$ indicates Experience TI at the time *t*; and $\Delta Experience_{t}$ indicates the increase value of Experience TI. α is a parameter indicating the *maximum increase value* of the experience. $max_{Experience}$ is a parameter indicating the *maximum value* of Experience TI (obviously $\alpha <max_{Experience}$). Usually it is more convenient for Experience TI to use the same scale with trust (i.e., the range of [0,1]), thus, $max_{Experience}$ is 1. Consequently, the Equations (4) and (5) can be rewritten as:
  (6) $$Experience_{t+1}=Experience_{t}+\alpha \times (1-Experience_{t})$$
  (7) $$\mathrm{or}\;Experience_{t+1}=\left(1-\alpha \right)\times Experience_{t}+\alpha$$
  As shown in the Equation (6), the *increase value* $\Delta Experience_{t+1}=\alpha \times (1-Experience_{t})$ is relatively large when the current value $Experience_{t}$ is small; but the *increase value* is reaching to 0 when the current value $Experience_{t}$ is high (approaching to 1).
- *Experience Decrease (in case of an uncooperative interaction occurs)*:
  The mathematical model for the Experience Decrease is as following:
  (8) $$Experience_{t+1}=Max\langle min_{Experience},\;Experience_{t}-\beta \times \Delta Experience_{t+1}\rangle$$
  where $\Delta Experience_{t+1}$ is specified as in Equation (2); and β is as a damping factor controlling the rate of the decrease. The β parameter can be fixed or dynamic depending on situations, but it should be always greater than 1 because the experience relationship is hard to gain but easy to lose. $min_{Experience}$ is a parameter indicating the minimum value of the experience (i.e., 0), which guarantees that the experience value cannot go lower than that.
- *Experience Decay (in case of no interaction)*:
  Experience TI decreases if there is no interaction during a period of time. However the rate of the decrease may vary according to the level of current status of the relationship (i.e., the current experience value). If the current status is high (meaning that there is a strong tie between two entities) then the decrease is not much; but if current status is low (i.e., a weak tie between the two) then the decrease is much. Hence, experience is assumed to require periodic maintenance but strong ties tend to persist longer even without reinforcing cooperative interactions. Decay is assumed to be inversely proportional to the current experience value; thus, experience with a high value will exhibit less decay than experience with a low value. Then, the mathematical model for the Experience Decay is proposed as following:
  (9) $$Experience_{t+1}=Max\langle min_{Experience},\;Experience_{t}-\Delta decay_{t+1}\rangle$$
  (10) $$\mathrm{where}\;\Delta decay_{t+1}=\delta \times \left(1+\gamma -\frac{Experience_{t-1}}{max_{Experience}}\right)$$
  The δ is a parameter indicating the *minimal decay value* of Experience which guarantees that even strong ties still get decreased if experience is not maintained. γ is a parameter indicating the *rate of decay* which can be fixed or dynamic depending on particular situations.

According to the Experience TI model, in order to obtain a high experience value (i.e., a strong tie between a trustor and a trustee), it is required to have many cooperative interactions in a short duration of time. And when it gets high, it is not easy to decay as time goes by. However, uncooperative interactions can highly damage the experience relationship, especially when the current state is not strong. This is similar to what happens in the real human world, thus, we believe the proposed Experience TI model can effectively migrate the experience relationship from human sociology environment to entities in the SIoT.

### 4.4. Reputation TI Evaluation Model

Reputation is a social concept which corresponds to what is generally understood about entity’s characteristics. Reputation of any entity should be public and is determined by aggregating opinions of other in its social groups. Reputation has been intensively carried out in both computer sciences and information sciences recent years [7,52,53,54]. A reputation system is frequently found in e-Commerce websites for encouraging online transactions by providing evidences of trust to help people interact with each other without having firsthand knowledge. Thus, in this case, reputation can serve as a basic for trust. Reputation systems are mostly based on feedback from the participants in the transactions (as the trustors) about how a trustee has accomplished a given task (the trust goal), in both positive and negative opinions. This feedback is then aggregated and presented to the public as an estimate of the trustee’ trustworthiness. Therefore, a reputation mechanism is necessary for managing feedback as well as for evaluating, propagating, and maintaining reputation values for each entity in SIoT. For instance, eBay, IMDb and Keynote use a centralized trust authority to establish and maintain user ratings whereas Google has developed a distributed approach for assessing reputation of webpages based on backlinks. They use several heuristic algorithms for reputation integration and update on evaluation process.

**Figure 9:**
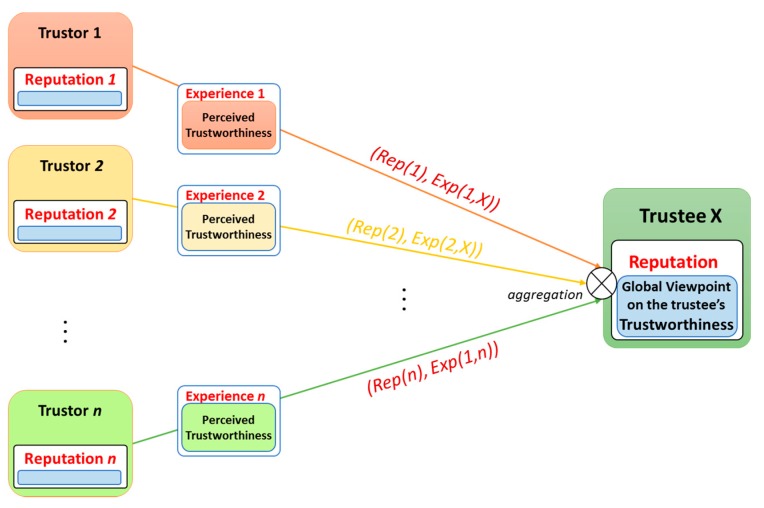
Conceptual Reputation Model incorporating the Experience concept.

In the scenarios of the SIoT environment, as mentioned in Section 4.3, feedback is a form of outcomes of interactions; and Experience TI is considered as an aggregation of feedback from a specific entity to another. Experience TI model shows that each of entities (as the trustor) which has previous interactions with a specific entity (as the trustee) holds an opinion about the trustee as its experiences. And if all of these entities share their opinions as recommendations about the trustee to others, we can come up with a model that aggregates these recommendations to form a unique value about the trustee as the trustee’s reputation. A necessary consideration is that each of the recommendations contributes differently to the trustee’s reputation. The weight a trustor’s recommendation contributing to the trustee’s reputation depends on both Experience TI (between the trustor and the trustee) and Reputation of the trustor itself. Therefore, appropriate reputation models should not only take the experience values into account but also the reputation values of the trustors (The reputation conceptual model is illustrated in Figure 9. It is reasonable because obviously, an entity with high reputation contributes more than an entity with lower reputation to the trustee’s reputation.

We have come up with a non-bias mechanism for calculating recommendation and reputation values of trust for all entities in a distributed network in [55]. The mechanism, however, is conducted in the centralized authority and it requires to aggregate necessary information about the social relationships of both trustors and the trustee. In this article, inspired by the PageRank^TM^ idea in [8], we have proposed a novel approach to calculate reputation values for entities over the SIoT networks. Two challenges appeared when designing a model for the Reputation TI based on the PageRank^TM^ algorithm: (i) Different weights of recommendations from many entities to a particular entity; and (ii) Recommendations could be both positive and negative; positive recommendations occur when Experience value Exp(i,N)>θ result in increasing reputation of the target entity N whereas negative recommendations (Exp(j,N)<θ) should reduce reputation. θ is the threshold parameter indicating whether an Experience is considered as negative or positive. The original PageRank^TM^ considers same weights for all links from a webpage to another and the mathematical model correctly works for only positive links’ values (the weights for all links are assigned as 1/N where N is the total number of webpages in a network).

A modification of the PageRank^TM^ model for the Reputation TI so-called Rep-Ranking is proposed as following:

(11) $$Rep_{Pos}\left(X\right)=\frac{\left(1-d\right)}{N}+d\times \left(\underset{\forall i}{\sum }Rep_{Pos}\times \frac{Exp\left(i,X\right)}{C_{Pos}\left(i\right)}\right);\;\forall i\;that\;Exp\left(i,N\right)>\theta$$

(12) $$Rep_{Neg}\left(X\right)=\frac{\left(1-d\right)}{N}+d\times \left(\underset{\forall i}{\sum }Rep_{Neg}\left(i\right)\times \frac{1-Exp\left(i,X\right)}{C_{Neg}\left(i\right)}\right);\;\forall i\;that\;Exp\left(i,N\right)<\theta$$

(13) $$Rep\left(X\right)=\mathrm{Max}\left(min_{Rep},\;Rep_{Pos}\left(X\right)-Rep_{Neg}\left(X\right)\right)$$

where:

- N is total number of entities in the networks for calculating Reputation
- $Rep_{Pos}\left(i\right)$ is called positive reputation of the entity *i* which considers only positive recommendations.
- $C_{Pos}\left(i\right)$ = $\underset{Exp\left(i,\;j\right)>\theta }{\sum }Exp\left(i,\;j\right)$ is the total values of all positive recommendations that the entity *i* is currently sharing.
- $Rep_{Neg}\left(i\right)$ is called negative reputation of the entity *i* which considers only negative recommendations.
- $C_{Neg}\left(i\right)$ = $\underset{Exp\left(i,\;j\right)<\theta }{\sum }\left(1-Exp\left(i,\;j\right)\right)$ is the total values of all complements of the negative recommendations that the entity *i* is currently sharing.
- Rep(i) is the reputation of the entity *i* that we are interested.
- $min_{Reputation}$ is a parameter indicating the minimal value of reputation (i.e., 0). This guarantee the reputation value will not go below the$\;min_{Reputation}$.
- Experience(i,X) is the Experience TI from the entity *i* toward the entity *X* described in Section 4.3.
- *d* is the damping factor. Various studies on PageRank-related literature have tested different damping factors for ranking webpages on the Internet, and they have come up with an appropriate value around 0.85. The research on the damping factor for the Reputation TI model is left as our future work.

Similar to PageRank^TM^, the Equations (8)–(10) form a normalized probability distribution of the reputations (positive reputation, negative reputation and overall reputation) after conducting a number of iterations throughout the network; as well as calculating and updating reputation values for all entities in the network after each iteration. Therefore, the reputation model can be implemented in a centralized system to calculate reputation values for all of entities in a social network. Details of the mechanism can be found in various related literature such as in [8,56,57,58]. This approach could face a critical challenge when the size of a network dramatically increases (i.e., millions of entities). However, by using classification machine learning algorithms with an appropriate semi-distributed architecture, whole social network can be divided into smaller sub-populations, resulting in the feasibility of conducting the proposed reputation model [59,60].

### 4.5. Aggregation Mechanism for REK Trust Evaluation Model

The outcome of the REK Trust Evaluation model is aggregated based on the triad Reputation, Experience, and Knowledge TIs. It also requires to aggregate TAs to derive Knowledge TI. As clarified in the conceptual trust model as well as the REK model, these aggregations should take both environmental factors and trustor’s propensity into consideration. Technically, there are two common approaches to attain TIs from associated attributes; and to finalize an overall trust value from the three TIs. The choice between the two depends on specific scenarios such as information modelling of TAs, of the trustor’s preferences, and of the environmental factors.

The first approach is to use mathematical models such as weighted sum [61,62], Bayesian neutron networks [63,64], and machine learning algorithms such as linear regression [65]. These models use mathematical models to express trustor’s propensity and environment conditions by assigning weights for individual features (i.e., TAs and TIs). These values can be autonomously updated depending on outcomes of the models by using a feedback mechanism. The second method makes use of an inference engine for inferring new knowledge from a knowledge-base such as reasoning mechanisms [66] and fuzzy-based mechanisms [18,67]. These inferring mechanisms are frequently used for deriving causal-consequence knowledge that is also appropriate for incorporating trustor’s propensity and environmental factors. In the second approach, all trust-related information already obtained (e.g., TAs, Experience TI, and Reputation TI) are represented in form of facts; trustor’s propensity and environmental factors are represented in form of logics applied upon the facts (e.g., rules in reasoning mechanisms, and membership functions in fuzzy-based mechanisms). Based on the set of logic, an inference engine can draw new knowledge that is being interested such as Knowledge TI and the overall trust value. In real implementation, a set of default logics should be already investigated and deployed for all entities. Then a trustor might have more preferences or a considering environment might have different conditions; then these factors are converted into logics that replace or supplement the default set of logics.

**Figure 10:**
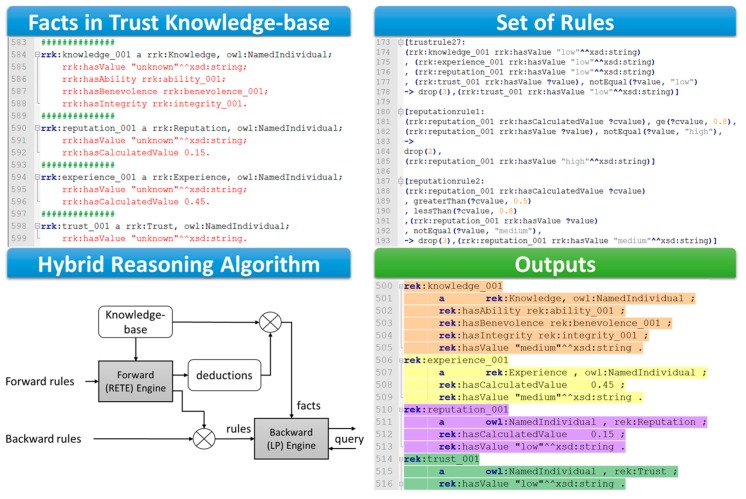
Reasoning mechanism used in a demonstration for inferring trust value in the REK trust model.

For example, we have used the Apache Jena framework in the trust demonstration for the User Recruitment in MCS use-case which integrates several types of inference engines including the generic rule-based reasoner that enables predefined rules. Before that, all TAs, Reputation TI, and Experience TI already obtained are converted into semantic information as metadata in forms of facts in Description Logics [68] represented in RDFS/OWL languages (Figure 10). The Jena integrated rule-based reasoner supports both forward chaining and tabled backward chaining reasoning strategies as well as the hybrid approach. For example, a generic hybrid reasoner in the Jena framework is used in the demonstration to infer reputation values and experience values in form of levels (i.e., *low*, *medium*, and *high*) from the actual calculated values (the calculated values are in the range [0–1] and obtained using the proposed Experience TI model and Reputation TI model); as well as to infer the level of trust which is the overall trust value we are interested.

In the User Recruitment in MCS demonstration, values of TAs such as Spatial Distance, Availability, Dependability, Cooperativeness and Honesty are already obtained and then represented in form of facts in the trust knowledge-based. Trustor’s propensity is represented in form of rules upon literals introduced in the facts. For example, with a same trustee with calculated reputation value is 0.45; a trustor could consider that Reputation TI is *low* but another trustor considers Reputation TI as *medium*. These kinds of preferences are represented using Jena syntax rules illustrated in Figure 10. Then a hybrid reasoner is used to derive the overall trust value as the level of trust (i.e., *low*, *medium*, and *high*). As illustrated in Figure 10, based on facts and set of rules, the reasoning engine infers the Reputation TI value as “*low*”, the Experience TI value as “*medium*” and the Knowledge TI value as “*medium*”. These inferred values are as new knowledge (new facts) in the Knowledge base, as a result, additional rules are triggered; new other facts are created. This process would iterate until a goal has reached or no rules can be matched (i.e., when the overall trust value (level of trust) is obtained). It is worth to note that different trustor profiles have different associated set of rules, resulting in different subjective level of trust inferred.

## 5. Conclusions and Future Work

In this article, we have provided a comprehensive understanding on trust concept in the SIoT with the REK evaluation model for trust which incorporates the three major TIs Reputation, Experience and Knowledge considering multi-dimensional trust aspects from direct observation to third-party information. We also have examined necessary TAs for covering the direct observation of trustworthiness as the Knowledge TI considering the three dimensions Ability, Benevolence and Integrity of any entities in the SIoT environment. We have also proposed prototypes for the Experience and Reputation TIs by proposing the associated mathematical models leveraging the sociological behaviors of human in the real world as well as the Google PageRank^TM^ ideas in the webpage ranking areas, respectively. Finally, we combine the TAs of the Knowledge TI, the Experience TI and the Reputation TI using Semantic-Web technologies for finalizing the overall trust value as the *level of trust*.

This article opens a large number of research directions in order to fulfil the trust evaluation platform. The first direction is to adapt the trust evaluation model to various scenarios and use-cases that require to figure out a set of TAs for Knowledge TI in detail as well as appropriate mathematical parameters for Experience and Reputation TIs.

The second direction could be a smart mechanism to reflect the trustor’s propensity and environmental factors to the trust evaluation model such as an autonomous weighted sum mechanism with machine learning for adaptively changes the weights according to a particular context. Another solution could be a smart rules generators for the trust knowledge-base so that the final trust value will be obtained in a context-awareness manner. In the demonstration in Section 4.5, the rules are predefined using understanding of a specific service with user preferences on trust. This can be improved by using machine learning techniques for rule pattern recognition in an automatic rule creation mechanism.

Another research direction could be the improvement of the reasoning mechanism so that it can autonomously adapt with changes of the knowledge base, resulting in an autonomous trust computation framework and with real-time data streaming (stream reasoning). The usage of Semantic Web technologies such as the Ontology, RDFS and reasoning mechanism could also be improved for more complex use cases and for the support of real-time processing and scalability.

Final direction could be other mathematical models for the Experience and Reputation TIs which not only base on intensity and outcomes of interactions but also other complicated features extracted from particular contexts such as features of mutuality or difference in social environment.

## Figures and Tables

**Table 1:** Some keywords of trustworthiness from trust-related literatures classified into three dimensions.

Ability TAs	Benevolence TAs	Integrity TAs
Competence, ability, capability, expertness, credibility, predictability, timeliness, robustness, safety, stability, scalability, reliability, dependability	Good intention, goodness, certainty, cooperation, cooperativeness, loyalty, openness, caring, receptivity, assurance	Honesty, morality, completeness, consistency, accuracy, certainty, availability, responsiveness, faith, discreetness, fairness, promise fulfilment, persistence, responsibility, tactfulness, sincerity, value congeniality, accessibility
